# Neural Plastic Effects of Working Memory Training Influenced by Self-perceived Stress in Stroke: A Case Illustration

**DOI:** 10.3389/fpsyg.2016.01266

**Published:** 2016-08-30

**Authors:** Ada W. S. Leung, Lauren M. Barrett, Darcy Butterworth, Karin Werther, Deirdre R. Dawson, E. Sharon Brintnell

**Affiliations:** ^1^Department of Occupational Therapy, University of AlbertaEdmonton, AB, Canada; ^2^Neuroscience and Mental Health Institute, University of AlbertaEdmonton, AB, Canada; ^3^Rotman Research Institute, Baycrest Health SciencesToronto, ON, Canada; ^4^Department of Occupational Therapy, Glenrose Rehabilitation HospitalEdmonton, AB, Canada; ^5^Department of Occupational Science and Occupational Therapy, University of TorontoToronto, ON, Canada

**Keywords:** stroke, self-perceived stress, working memory training, fMRI, neuroplasticity

## Abstract

This case study examined the effects of auditory working memory (WM) training on neuroplastic changes in stroke survivors and how such effects might be influenced by self-perceived stress. Two participants with a history of stroke participated in the study. One of them had a higher level of self-perceived stress. Both participants underwent a course of auditory WM training and completed baseline and post-training assessments such as self-perceived stress, performance satisfaction questionnaires, behavioral task performance, and functional magnetic resonance imaging. They were trained on a computerized auditory WM task (*n*-back) 5 days a week for 6 weeks, for a total of 20 h. Participant 1 had high levels of perceived stress, both pre- and post-training, and showed improvement on the satisfaction aspect of functional engagement only. Participant 2 had lower levels of perceived stress and demonstrated improvements on all performance tasks. Neuroimaging results showed evidence of improved neural efficiency on the trained task for participant 2. The results shed light on the need to evaluate psychological influences, e.g., stress, when studying the neuroplastic changes in people with stroke. However, the case design approach and other factors that might have positively influenced outcomes mean that these results must be interpreted with a great deal of caution. Future studies using a larger sample are recommended to verify the findings.

## Introduction

Working memory (WM) is considered one of the most important cognitive components for stroke rehabilitation (Cicerone et al., 2011). WM is defined as the capacity to maintain and manipulate information for a period of several seconds (Baddeley, 1986).

The linkage between stress and cognition can be drawn from the cognitive activation theory of stress (Ursin and Eriksen, 2004). Stress response activates when there is a discrepancy between what really happens and what is expected (Ursin, 2009). An individual evaulates whether a situation is stressful based on prior experience, and his/her response to stress can be either positive (i.e., effective coping mechanisms), negative (i.e., feeling of hopelessness), or uncertain (i.e., feeling of helplessness; Levine and Ursin, 1991). Thus, the level and duration of stress can increase if the individual has disorted expectations and weak coping mechanisms. Sustained stress has many pathological effects; a well-known effect is the excessive exposure of glucocorticoid, which has adverse effects in the hippocampus, a structure vital to learning and memory (Sapolsky, 1996).

The relationship between stress and WM has been shown in recent studies. For example, stress exposure from environmental disasters is shown to impaire WM performance (Li et al., 2015). Individuals exposed to prolonged academic stress are found to have positive correlation between self-perceived stress scores and brain electrical activities on WM tasks (1- and 2-back tasks; Yuan et al., 2015). Some researchers show that WM training improves WM performance in healthy adults with stess (Gavelin et al., 2015). Furthermore, a behavioral study has found improvements of cognitive function after working memory training in patients with stroke, but the study did not include mesaures for self-perceived stress (Westerberg et al., 2007). Hence, how stress affects the neuroplsatic changes after WM training has yet to be addressed.

In healthy adults, neuroplastic changes associated with WM training have been widely examined using *n*-back tasks and functional magnetic resonance imaging (fMRI; e.g., Klingberg, 2010; Schneiders et al., 2012). With identical paradigms, auditory WM tasks appear to be more difficult than their visual counterparts (Jaeggi et al., 2010), providing an added value for using auditory *n*-back tasks as training. Auditory WM tasks typically activates the inferior and middle frontal gyri, middle and superior temporal gyri, and superior and inferior parietal lobes (Alain et al., 2001; Owen et al., 2005). With training, most studies reported decreased neural activations in the inferior, superior and middle frontal gyri and the inferior and superior parietal lobes (Garaven et al., 2000; Landau et al., 2004). Activation decreases reflect more efficient neural processing, i.e., the same task can be performed with fewer cognitive resources compared to pre-training conditions (Kelly and Garavan, 2005). Some studies reported activation increase in frontal and parietal regions after training, suggesting a redistribution of neural networks that facilitate skill transfer (Olesen et al., 2004; Kelly and Garavan, 2005; Westerberg and Klingberg, 2007).

Although, previous studies have shown that WM training is effective for improving cognitive performance (e.g., Westerberg et al., 2007), some studies have reported that WM training does not transfer to other tasks or skills (e.g., Shipstead et al., 2012; Melby-Lervåg and Hulme, 2013). For studies demonstrating positive outcomes, a training period of 5 weeks was commonly used to achieve neuroplastic effects on *n*-back tasks (Klingberg, 2010). Another feature in WM training is adaptive procedures in which the task difficulty co-varies with performance (von Bastian et al., 2013). Some studies used non-adaptive procedures where task difficulty was controlled (e.g., Li et al., 2008; Leung et al., 2014).

### Objectives

This case study examined the effects of stress on the neuroplastic changes of WM training in two stroke survivors.

## Background

Two participants with a history of stroke and demonstrated memory problems in daily activites were recruited from an out-patient clinic. The participants had difficulty with WM as indicated by a score of 3 or above on WM items of the Cognitive Failure Questionnaire (Broadbent et al., 1982). They were not receiving any other therapies during the time of experiment and were fitted for fMRI. Both participants gave written informed consent. The study was approved by the Health Research Ethics Board of the University of Alberta (UA).

Participant 1 was 37-year-old man and suffered a mild stroke involving the right middle cerebral artery (MCA) 2 years before the study. He also had the stroke 20 years ago. He had completed a bachelor degree in a local college and was working full time in a private company as a secretary. He was single, lived alone, and had no documented history of any other neurological or psychiatric conditions. He reported memory deficits that were noticeable during work and social interaction with colleagues.

Participant 2 was 37-year-old woman and suffered a right-sided cerebral infarction involving the right MCA 4 years before the study. She had also sustained two episodes of brain-stem cerebral infarction during hospitalization for her stroke. She had completed post-secondary education and was working full time as a managerial assistant in a company. She was single and lived alone. Her job required her to interact with people, and she reported having difficulties with concentration and memory which affected her work performance.

### Behavioral assessments and results

*Perceived Stress Scale (PSS)* is a commonly used psychological instrument for measuring the perception of stress (Cohen et al., 1983). The psychometric properties of PSS have been confirmed (Lee, 2012). PSS has been used to assess perceived stress in people with stroke (Ostwald et al., 2008; Santos et al., 2015). The questions in PSS asked about participants' feelings and thoughts during the last month, and how often they felt a certain way. All of the 10 items were used in this study. PSS scores were obtained by reversing responses (e.g., 0 = 4 points, 1 = 3 points) to the four positively stated items, and then summing the scores across all items. Higher score indicated higher stress level.

*Canadian Occupational Performance Measures (COPM)* identifies functional difficulties that are unique to an individual (Law et al., 2005). Participants reported their satisfaction and performance level on activities of their choice on an ascending 10-point scale. An increment of two or more points on either scale over time has been shown to be clinically significant (Law et al., 2005).

Test scores at baseline were shown in Table 1. Participant 1 showed a higher level of self-perceived stress (PSS = 27/40) than participant 2 (PSS = 13/40). Both participants had comparable performance scores (COPM performance scale = 5.3 and 5.5 for participant 1 and 2, respectively), but participant 2 was more satisfied with her task performance than participant 1 (COPM satisfaction scale = 1.7 and 5.3 for participant 1 and 2, respectively).

**Table 1:** **Results of PSS and COPM scores and the performance of 1-back and 2-back tasks during fMRI (3-back was not performed during fMRI)**.

		**Participant 1**		**Participant 2**	
		**Pre**	**Post**	**Pre**	**Post**
PSS	Total score	27/40	33/40	13/40	11/40
COPM^a^	Mean performance (P)	5.3/10	5.3/10	5.5/10	7.3/10
	Mean satisfaction (S)	1.7/10	4.0/10	5.3/10	8.3/10
N-back performance during fMRI	1-back hit rate (%)	94.2	96.1	97.8	100.0^*^
	1-back RT (ms)	567.9	669.4	691.1	605.9
	2-back hit rate (%)	95.4	97.2	95.6	97.8^*^
	2-back RT (ms)	599.9	674.5	897.0	771.0

*RT, reaction time; Pre, pre-training testing; Post, post-training testing*.

* *Data from two instead of all three fMRI runs as one of the runs was contaminated due to technical problems of the MRI scanner*.

a *Details of the goals and ratings (P, performance; S, Satisfaction): Participant 1 had three goals: (1) attend to tasks at work (Pre: P = 6, S = 2; Post: P = 6, S = 3); (2) able to stay on conversation with others (Pre: P = 5, S = 1; Post: P = 5, S = 6); and (3) do multiple things at once (Pre: P = 5, S = 2; Post: P = 5, S = 3). Participant 2 had four goals: (1) attend details in tasks (Pre: P = 6, S = 6; Post: P = 7, S = 8); (2) organize appointments (Pre: P = 6, S = 6; Post: P = 8, S = 9); (3) stay on tasks without being distracted (Pre: P = 5, S = 4; Post: P = 7, S = 8); and (4) multitasking (Pre: P = 5, S = 5; Post: P = 7, S = 8)*.

### Training tasks and schedule

The training tasks consisted of a series of auditory *n*-back tasks, *n* = 1, 2, and 3, created using E-prime2.0 (Psychology Software Tools). Participants monitored a series of auditory stimuli (letters and digits) through headphones. They had to press a button when a stimulus was identical to the one presented one trial back (1-back), 2 trials back (2-back), or 3 trials back (3-back). Stimuli were arranged in blocks and presented in a random sequence.

The training sessions occurred 5 days a week for 6 weeks. Each day, participants completed four tasks (10 min each) at home. There was a gradual progression of task difficulty, meaning that there were more 1-back than 2- or 3-back tasks in week 1 and more 3-back than 1- or 2-back tasks in week 6. The entire training program consisted of 40 blocks for each type of task. This training schedule controlled the amount of exposure to each type of task (Li et al., 2008; Leung et al., 2014). A student met with the participants once every week to give feedback on their performance.

### fMRI scanning

Participants performed 1-back and 2-back tasks in an fMRI session before and after training. From prior studies, Leung et al. (2014) and Leung and Alain (2011), 3-back tasks were difficult for patients and some healthy adults, which reduced the reliability of neuroimaging results. Therefore, 3-back tasks were not administered in fMRI. There were three runs for each session, each containing six blocks of tasks, with each block followed by a 30-s rest period. Each task block lasted 40 s and consisted of 20 trials, and was preceded by 2 s of spoken instruction, indicating 1-back or 2-back task. Each trial consisted of an auditory stimulus lasting 600 ms and a pause period lasting 1400 ms. Participants pressed a button whenever they heard a target. The 1-back and 2-back tasks were randomly distributed with a total of nine blocks for each task.

The fMRI scanning was performed at the Peter S. Allen MR Research Center at UA. A 1.5-T MRI system (Siemens) with a standard birdcage head coil was used. Structural T1 weighted anatomical volumes were obtained (axial orientation, TR = 2080 ms, TE = 4.38 ms, FOV = 256 mm, slice thickness = 1 mm). T2^*^ functional images were obtained using EPI acquisition (TR = 1950 ms, TE = 40 ms, flip angle = 90°, FOV = 256 mm, effective acquisition matrix = 64 × 64). Each functional sequence consisted of 36 4-mm thick axial slices, positioned to image the whole brain with a duration of 7 min and 42 s.

### Overall procedure

Participants attended an intake interview at UA. They were assessed on PSS and COPM, and were given a hearing test to ensure they had a normal range of hearing thresholds in both ears. They performed the pre-training fMRI testing on 1-back and 2-back tasks. After that, they performed the training at home using a laptop provided by the researchers. After training, participants repeated the fMRI scanning and behavioral assessments. A research assistant not involved in the training administered the behavioral assessments.

### Analysis of behavioral data

The total scores of PSS were reported. The mean scores of the performance and satisfaction scales of the COPM were calculated based on participants' goals (Law et al., 2005). Hit rate and reaction time of *n*-back tasks were extracted. The training data was presented by dividing the mean hit rate and reaction time obtained from each week by that of week 1.

### Analysis of fMRI data

Preprocessing was performed using Statistical Parametric Map (SPM8) (Wellcome Department of Cognitive Neurology, Institute of Neurology, UK), running under Matlab 2012a (MathWorks, MA, USA). The first five images of each run were removed to avoid unstable signals. The remaining images of each run underwent a slice timing procedure to correct for slice sequence. The images were re-aligned with the first image for motion correction and co-registered with high-resolution structural images. The images were then spatially normalized and resliced to the MNI standard template (Montreal Neurological Institute). The images were spatially smoothed by convolution with a three-dimensional Gaussian kernel (FWHM = 8 mm).

The pre-processed data were modeled as a general linear model (GLM) using separate regressors for the sustained and transient activities (Alain et al., 2008). The GLM model consisted of 10 regressors which included both pre- and post-training data, each containing two types of blocks (1-back and 2-back as sustained activity), two types of targets (hit response of 1-back and hit response of 2-back as transient activity), and a rest condition. The time course of the neural activity was modeled using the canonical hemodynamic response function. The resulting time series data was high-pass filtered with a threshold of 128 s to remove low-frequency drift.

The first-level analysis was done by setting up *T* contrasts for pair-wise comparisons. All contrasts were performed on sustained activities (i.e., task blocks) to minimize neural activations elicited by motor responses. A spatial cluster extent threshold was applied by using AlphaSim with 1000 Monte Carlo simulations to control for multiple comparisons. This procedure yielded a minimum cluster size of 196 μl (correspond to 3 voxels in the original acquisition space), with a map-wise false-positive probability of *p* < 0.03 (Alain et al., 2008). Hence, only significant activations that had a cluster size of 196 μl and *p* < 0.01 (corrected) for each voxel were reported. Activation maps were presented using the MRIcron program (http://www.sph.sc.edu/comd/rorden/mricro.html), and the regions of activation were identified by Automated Anatomical Labeling (AAL) on the standard MNI template (Tzourio-Mazoyer et al., 2002).

## Results

Both participants completed 6 weeks of training. Both of them showed improvement on the satisfaction scale of the COPM, and participant 2 showed additional improvement on the performance scale (see Table 1 for the goals and the corresponding ratings).

Regarding perceived stress, participant 1 showed higher scores on PSS compared to participant 2 and an increase of PSS score from the pre-training to the post-training testing (Table 1). Participant 2 showed a stress level comparable to norm samples (Cohen and Williamson, 1988), and the PSS scores were similar in both the pre- and post-training testing.

**Figure 1:**
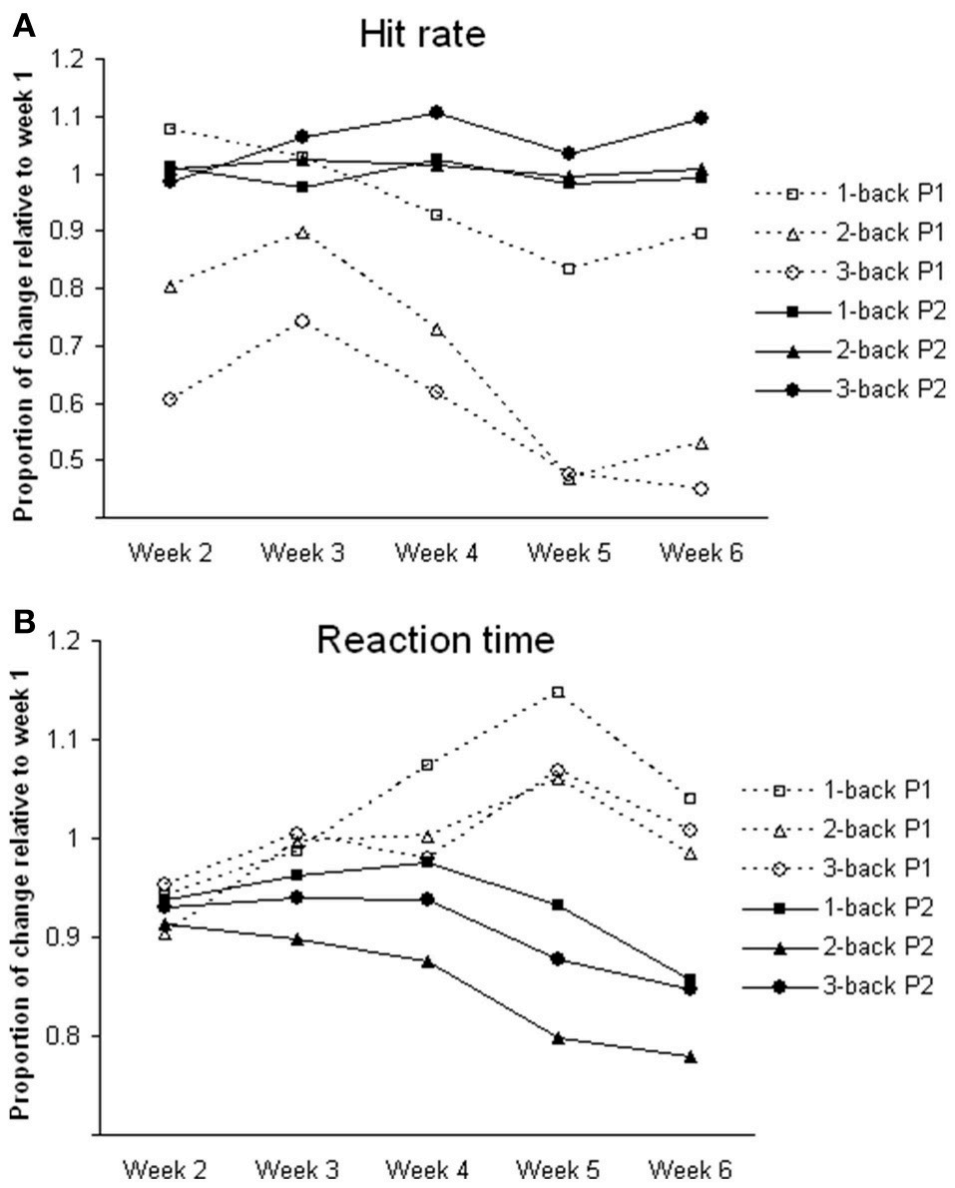
**Changes of task performance during training**. All values are relative to week 1. **(A)** Hit rate; calculated by dividing hit rate (%) in each week by that in week 1; greater the value better the performance. **(B)** Reaction time; calculated by dividing reaction time (ms) in each week by that in week 1; greater the value slower the reaction time. P1, participant 1; P2, participant 2.

For *n*-back tasks, both participants showed comparable hit rates and reaction times during fMRI scanning (Table 1). However, during training, participant 1 showed a gradual decline in hit rate and response speed (i.e., longer reaction time) in all of the *n*-back tasks over the 6-week period. Participant 2 showed improvement in hit rate, especially during the 3-back task, and reaction time across weeks (Figure 1).

**Figure 2:**
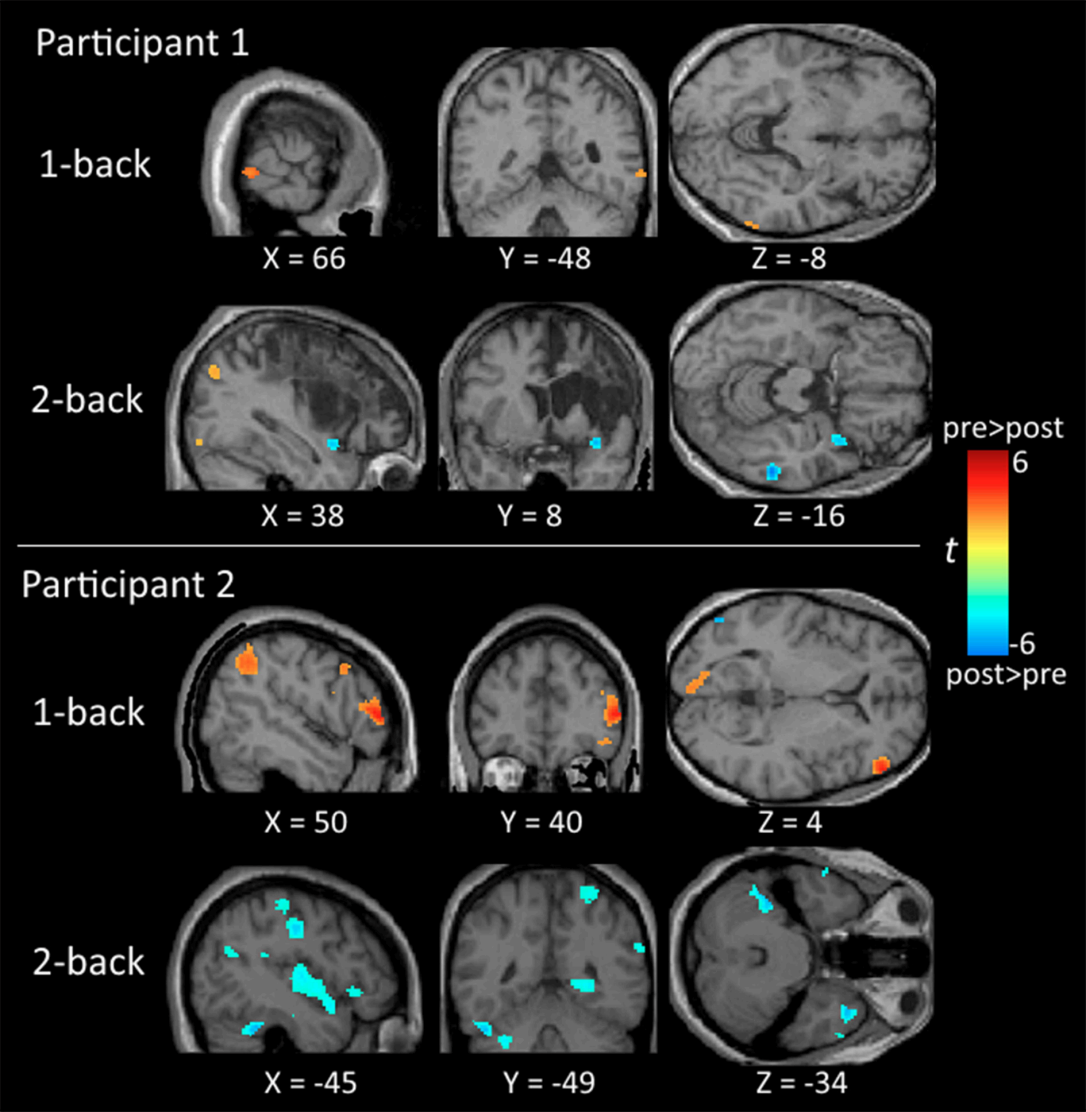
**Brain maps comparing the neural activation between the pre-training and post-training testing**. All activations are significant at *p* < 0.01 (corrected) and cluster size >196 μl.

Regarding neuroimaging findings, participant 1 showed significant activation in the middle temporal gyrus and inferior parietal lobe for the 1-back and 2-back tasks before training, and in the middle temporal gyrus after training. There was no indication of significant frontal activities for participant 1. For participant 2, there was significant activation in the frontal and parietal regions in the 1-back task before training, and the activation subsided after training. In the 2-back task, there was significant activation in the frontal regions before training and substantial activation in the middle temporal gyrus, inferior parietal lobe, and cerebellum after training (Table 2 and Figure 2).

**Table 2:** ***T* contrasts comparing the pre-training and post-training activation**.

			**MNI coordinates**						
**Brain regions**			**L/R**	**BA**	***x***	***y***	***z***	***T***	**voxel**
**PARTICIPANT 1**									
1-back	Pre > Post	Middle temporal gyrus	R	37	66	−52	−4	3.78	162
	Post > Pre	Middle temporal gyrus	L	20	−56	−36	−12	8.41	96
2-back	Pre > Post	Middle temporal gyrus	R	37	60	−60	8	3.25	20
		Inferior parietal lobe	L	40	−54	−34	50	2.85	64
	Post > Pre	Middle temporal gyrus	R	20	58	−40	−14	3.99	55
**PARTICIPANT 2**									
1-back	Pre > Post	Middle frontal lobe	R	6	36	2	60	4.00	267
		Inferior frontal gyrus	R	45	50	40	6	4.21	292
		Middle temporal gyrus	R	21	68	−46	−4	3.13	42
		Inferior parietal lobe	L	7	−26	−64	44	3.38	130
			R	40	54	−48	42	3.68	305
2-back	Pre > post	Middle frontal gyrus	L	45	−42	30	26	4.35	957
			R	47	42	48	−10	3.44	729
	Post > pre	Middle temporal gyrus	L	20	−38	14	−34	4.08	143
		Inferior parietal lobe	L	40	−56	−48	40	3.30	137
			R	40	52	−50	40	3.51	191
		Cerebellum_Crus2	R		46	−74	−46	3.12	21

*All activations are significant at p < 0.005 and cluster size >196 μl. Only true activations are listed. BA, Brodmann's Area; L, left; R, right*.

## Discussion

Neural activities were examined in two participants who had different levels of self-perceived stress and self-perception of task performance and satisfaction. Overall, participant 2 showed favorable outcomes (i.e., reduced self-perceived stress, improved performance and satisfaction on functional tasks, and improved *n*-back performance). In contrast, participant 1 had a high level of self-perceived stress over the training period and showed improvement only on the satisfaction scale of the COPM. Neuroimaging findings showed differences between the two participants. While neural activation in the fronto-parietal regions, core regions for WM, was decreased for participant 2, there was no such activation in participant 1 in both the pre- and post-training testing.

For participant 2, the decrease of fronto-parietal activation was consistent with previous training studies in healthy adults (Garaven et al., 2000; Landau et al., 2004) and stroke patients (Leung et al., 2014). This suggests that the cognitive training implemented for this participant result in increased neural efficiency, as the process of updating and storage becomes more efficient and consequently requires less effort during the training (Kelly and Garavan, 2005).

Participant 1 did not appear to show frontal activities which are crucial for *n*-back tasks. He also performed poorly during the training, with decreased task accuracy and increased response time across weeks. This pattern was different from that of participant 2 as well as the findings of a patient (KB) reported in another study (Leung et al., 2014). In that study, KB was a 39-year old man and had multiple strokes damaging both the frontal and parietal regions. KB demonstrated improvements in all WM tasks throughout a 7-week auditory WM training and a pattern of neural activation resembled that of participant 2 in this study. Stress was not a concern for KB. Instead, his wife was very supportive, and he had a very happy family life. Past studies on healthy adults have shown that high-stress individuals elicited reduced neural activities during cognitive processing compared to low-stress individuals (Hayashi et al., 2012; Koric et al., 2012). Also, a review has found that even mild acute uncontrollable stress can cause a rapid and dramatic loss of frontal cognitive abilities, and prolonged stress exposure can cause structural changes in prefrontal dendrites (Arnsten, 2009). Hence, the fronto-parietal network in participant 1 might have been altered because of the higher level of stress experienced by the participant. In addition, participant 1 had a higher PSS score in the post-test and a decline of performance throughout the training but normal performance during the post-training fMRI testing. As the participant reported, the reason might be that the participant performed the training tasks during mid-night when he was tired and felt frustrated on the tasks. Previous clinical findings have shown that higher perceived stress measured by PSS was associated with less functional independence in people with stroke (Santos et al., 2015). In addition, high-stress level assessed by psychological questionnaires has been shown to correlate with high stress-induced cortisol responses which increase medial temporal activity (Henckens et al., 2016).

Our results show different patterns of neuroplastic changes in two participants with different levels of self-perceived stress. However, with only two participants, it is difficult to conclude how stress modulates the neural activity. Instead, our results shed light on the need to evaluate self-perceived stress when studying the neuroplastic changes in people with stroke. The results also suggest that stress could be one of the modulating factors influencing neuroplastic changes and behavioral outcomes of the training.

## Limitations

The case design approach and other factors that might have positively influenced outcomes mean that the findings must be interpreted with a great deal of caution. Additionally, a lower rating of the satisfaction scale of COPM in participant 1, compared to participant 2, suggests that additional psychological factors, other than self-perceived stress, may contribute to the differences in the results between the two participants.

## Concluding remarks

Two participants completed a course of auditory WM training. The participant with a high level of self-perceived stress performed worse on task performance and demonstrated less effective neuroplastic patterns compared to the other participant. The results highlight the need to assess self-perceived stress when studying training effects in people with stroke. Also, the results must be interpreted with caution, and future studies using a larger sample are recommended to replicate the findings.

## Author contributions

AL conducted the study, analyzed and interpreted the data, and wrote and revised the manuscript. LB conducted the study, analyzed the data, and wrote the draft of the manuscript. DB and KW recruited participants, performed screening to ensure the participants are fit for the study, and helped write the draft of the manuscript. DD and EB provided consultation, helped analyze and interpret the data, and revised the draft of the manuscript.

### Conflict of interest statement

The authors declare that the research was conducted in the absence of any commercial or financial relationships that could be construed as a potential conflict of interest.
